# A Novel Scoring System for Rupture Risk Stratification of Intracranial Aneurysms: A Hemodynamic and Morphological Study

**DOI:** 10.3389/fnins.2018.00596

**Published:** 2018-09-05

**Authors:** Pengjun Jiang, Qingyuan Liu, Jun Wu, Xin Chen, Maogui Li, Zhengsong Li, Shuzhe Yang, Rui Guo, Bin Gao, Yong Cao, Shuo Wang

**Affiliations:** ^1^Department of Neurosurgery, Beijing Tiantan Hospital, Capital Medical University, Beijing, China; ^2^China National Clinical Research Center for Neurological Diseases, Beijing, China; ^3^Center of Stroke, Beijing Institute for Brain Disorders, Beijing, China; ^4^Beijing Key Laboratory of Translational Medicine for Cerebrovascular Diseases, Beijing, China; ^5^Department of Bioengineering, School of Life Sciences, Beijing University of Technology, Beijing, China

**Keywords:** intracranial aneurysm, rupture, hemodynamic, morphological, computational fluid dynamics

## Abstract

**Objective:** The aim of the present study is to investigate the potential morphological and hemodynamic risk factors related to intracranial aneurysms (IAs) rupture and establish a system to stratify the risk of IAs rupture to help the clinical decision-making.

**Methods:** Patients admitted to our hospital for single-IAs were selected from January 2012 and January 2018. A propensity score matching was conducted to match patients. The morphological parameters were obtained from high solution CTA images, and the hemodynamic parameters were obtained in accordance with the outcomes of computational fluid dynamics (CFDs) simulation. Differences in the morphologic and hemodynamic parameters were compared. The significant parameters were selected to establish a novel scoring system (Intracranial Aneurysm Rupture Score, IARS). The comparison was drawn between the discriminating accuracy of IARS and the Rupture Resemblance Score (RRS) system to verify the value of IARS. Then, a group of patients with unruptured IAs was stratified into the high risk and low risk groups by IARS and RRS system separately and was followed up for 18–27 months to verify the value of IARS. The outcome of different stratifications was compared.

**Results:** The matching process yielded 167 patients in each group. Differences of statistical significance were found in aneurysm length (*p* = 0.001), perpendicular height (H) (*p* < 0.001), aspect ratio (AR) (*p* < 0.001), size ratio (SR) (*p* < 0.001), deviated angle (DA) (*p* < 0.001), normalized average wall shear stress (NWSSa) (*p* < 0.001), wall shear stress gradient (WSSG) (*p* < 0.001), low shear area ratio (LSAR) (*p* = 0.01), and oscillatory shear index (OSI) (*p* = 0.01). Logistic regression analysis further demonstrated that SR, DA, NWSSa, LSAR, and OSI were the independent risk factors of IAs rupture. SR, DA, LSAR, and OSI were finally selected to establish the IARS. Our present IARS showed a higher discriminating value (AUC 0.81 vs. 0.77) in comparison with the RRS (SR, NWSSa, and OSI). After follow-up, seven patients were subject to IAs rupture. 5/26 in high risk group stratified by IARS, yet 7/57 in high risk group stratified by RRS. The accuracy of IARS was further verified (19.2% vs. 12.3%, AUC for the IARS and the RRS was 0.723 and 0.673, respectively).

**Conclusion:** SR, DA, NWSSa, LSAR, and OSI were considered the independent risk factors of IAs rupture. Our novel IARS showed higher accuracy in discriminating IA rupture in comparison with RRS.

## Introduction

Intracranial aneurysms threaten as much as 2–3.5% of the population worldwide and are the leading cause of non-traumatic SAH (Müller et al., 2013). Thus, IAs could cause a devastating outcome with high mortality (50%) (Cross et al., 2003). Accurate discrimination of IAs prone to rupture and optimal treatment decision-making now still pose a great challenge to the clinical practicers (Thompson et al., 2015). The CFDs analysis has been demonstrated be a useful method to assist clinical strategy of cerebral vascular diseases in recent years. Several hemodynamic and morphological parameters have been demonstrated to be the independent risk factors for IAs rupture (Xiang et al., 2011, 2014; Meng et al., 2012; Paliwal et al., 2017). Until now, several stratification models have tried to establish risk of IAs rupture based on morphological and hemodynamic models (Dhar et al., 2008; Xiang et al., 2011). An effective system called rupture resemblance score (RRS) system [SR, normalized wall shear stress (NWSS), and OSI were included] was proposed recently by Meng et al. through the combination of the hemodynamic and morphological features of ruptured IAs (Xiang et al., 2011, 2015, 2016). Yet results from recent studies suggested that WSS might be strongly collinearity with the morphological characteristics (including AR). Besides, some other parameters including LSAR and WSSG were also correlated with IAs rupture closely (Fan et al., 2015; Zhang et al., 2016a,b; Levitt et al., 2017; Sano et al., 2017; Skodvin et al., 2017a; Varble et al., 2017). Given these, we assumed that higher discriminating accuracy could be achieved in discriminating ruptured from unruptured IAs.

The aim of this study is to identify the independent risk factors related to IAs rupture and establish a more comprehensive scoring system for rupture risk stratification of IAs using hemodynamic-morphological analysis, and to verify the effectiveness of our novel scoring system by comparing the value with previous scoring system.

## Materials and Methods

### Patient Selected and Inclusion/Exclusion Standards

This study retrospectively reviewed the patients admitted into our hospital for ruptured IAs between January 2012 and January 2018. The patients were selected in accordance with the standards as follows: Inclusion criteria (1) just had one IA, (2) had complete clinical records and follow-up data, (3) intracranial bleeding was identified by CT scan within 24 h after bleeding, and (4) nearby CTA was conducted 20-120 days before bleeding. Exclusion criteria: (1) patients were related to other intracranial tumor, angiostenosis and angio-malformation, including arteriovenous malformation, cavernous malformation, etc. and (2) CTA data was not suitable for morphological analysis or hemodynamic analysis.

### Propensity Score Matching

To exclude known clinical risk factors and balance the baseline, a propensity score matching (PSM) was conducted to match patients using STATA (12SE, Stata corporation, United States) based on database of patients with unruptured IAs in our institution. The propensity scores were calculated by using a logistic regression model consisting of the input variables: gender, age, hypertension, atherosclerosis, ever smoker, and IAs’ location. The matching rate was 1:1 for ruptured IAs to unruptured IAs. The final matched group was confirmed as appropriately matching using χ^2^ testing to validate equivalence of individual variables between each group.

### Vascular Modeling

The dicom data of last CTA, conducted before bleeding, were collected from the high solution CTA work station (Siemens, Berlin, Germany) and converted into slice dicom data (about 0.5 mm per slice). The dicom data were introduced into Mimics 17.0 (Mimics Research 17.0, Materialize, Belgium) and reconstructed for further study.

### Radiological Measuring and Morphological Parameter

**FIGURE 1:**
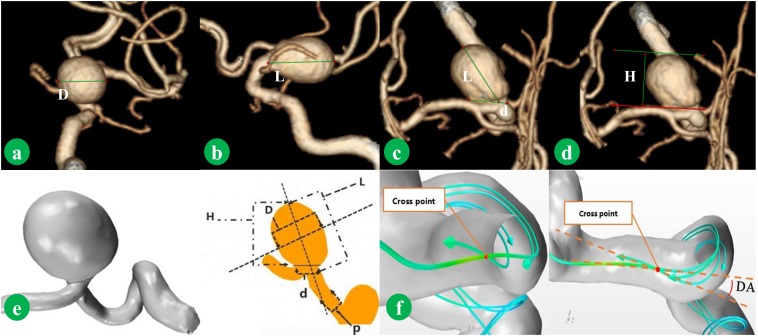
Morphological parameters were measured from CTA image. D is the maximum diameter of the body **(a)**. L is the maximum distance of the dome from the neck plane **(b)**. d is the average diameter of the neck **(c)**. H is the maximum perpendicular distance of the dome from the neck plane **(d)**. The method of measurement is shown in **(e)**. p is average diameter of the parent artery. Normal vector was combined. The deviated angle (DA), which was between co-velocity and normal vector, was measured **(f)**.

Radiological measuring was performed by two experience neurosurgeons (PJ and JW) using high solution CTA. Maximum length (L), maximum diameter of body (D), diameter of neck (d), perpendicular height (H), diameter of parent artery (P), and volume are measured from CTA as shown in **Figures 1a–e**. H is the maximum perpendicular distance of the dome from the neck plane. L is the maximum distance of the dome from the neck plane. d is the average diameter of the neck. p is average diameter of the parent artery. D is the maximum diameter of the body. Those parameters were measured twice, and the average was taken. AR, SR, undulation index (UI), ellipticity index (EI), and nonsphericity index (NSI) were calculated according to previous study (Dhar et al., 2008).

### CFD Simulating and Hemodynamic Parameter

To create 4–5 million units of finite tetrahedral and prism elements, each aneurysm model was meshed by STAR-CCM (STAR-CCM+ 12, Siemens, Germany). Then, the models were introduced into STAR-CCM fluid workstation (STAR-CCM+ 12, Siemens, Germany). In line with previous study (Xiang et al., 2011), incompressible Navier–Stokes equation served as the solver under pulsatile blood condition. When the patient enters the operating room, we use ultrasound to record the pulsatile waveform of the internal carotid artery. The pulsatile waveform was obtained by using transcranial Doppler ultrasound device on a representative patient with its magnitude scaled to the desired mean flow rate and was plotted to pulsatile curve. Blood was assumed as a Newtonian fluid with density ρ = 1056 kg/m^3^ and viscosity μ = 0.0035 Poise. Pulsatile curve served as the velocity inlet boundary condition, and free boundary condition was implemented at outlet. Four pulsatile cycles were simulated. The last cycle was yielded for the further study.

For each model, the normal vector of the vascular wall (the direction of flow was positive direction) was first reconstructed. The diverse-directed velocities were combined into a co-velocity as shown in **Figure 1f**. The angle between the normal vector and co-velocity was measured and defined as DA. Pressure maximum (Pm), pressure average (Pa), WSS maximum (WSSm), and WSS average (WSSa) were obtained from IA region, and parent Pa (pPa) and parent WSS average (pWSSa) were obtained from parent artery region. The WSS average and pressure average at peak of the systolic phase were applied for the further study. Low WSS was defined as less than 10% of WSS of parent artery (Xiang et al., 2011). The NPa, NWSSa, NPm, and NWSSm were calculated in line with the equations (1, 2, 3, and 4), respectively. OSI and RRT were calculated according to previous study (Dhar et al., 2008). OSI and RRT were the average over the dome area.

(1) $$\mathrm{NPa}=\frac{\mathrm{Pa}}{\mathrm{pPa}}$$

(2) $$\mathrm{NWSSa}=\frac{\mathrm{WSSa}}{\mathrm{pWSSa}}$$

(3) $$\mathrm{NPm}=\frac{\mathrm{Pm}}{\mathrm{pPm}}$$

(4) $$\mathrm{NWSSm}=\frac{\mathrm{WSSm}}{\mathrm{pWSSm}}$$

### Patients Follow-Up and Validation

To verify the accuracy of our stratification system, a group of other patients with unruptured IAs was followed up, whose aneurysms were found unintentionally. The patients who visited in the outpatient from January 2016 to October 2016 were enrolled. Those patients were followed up for 18–27 months by clinical visiting. To identify the change of IAs, CTA was conducted for every 4–6 months for patients with aneurysm under high rupture risk, and CTA was conducted for every 5–6 months for patients with aneurysm under low rupture risk.

Another PSM executed. Propensity scores were calculated for ruptured and unruptured IAs. A group of patients were selected in line with gender, age, hypertension, atherosclerosis, and ever smoker, and then stratified by IARS and RRS system, respectively. We matched the ruptured IAs to the unruptured IAs using a 1:9.

### Statistical Analysis

Measurement variables were compared by performing the chi-square test or Fisher’s exact test. Continuous variables were firstly assessed visually by performing the P-P plots and the Shapiro–Wilk test and then compared by performing the independent samples *t*-test. The ROC curve analysis was conducted for significant parameters on univariate analyses. Highest Youden index was employed to find the appropriate cut-off value. The results are presented in 95% confidence intervals. Intercorrelations between parameters were examined using Person correlation. To clarify the independent risk factors related to the rupture statues, multivariate regression analyses were conducted. ROC analyses were performed for the follow-up patients based on the IARS and the RRS, and the AUC were compared. The ruptured rate in high risk group and the unruptured rate in low risk group were also compared. A *p*-value of <0.05 was assumed to be of statistical significance. The result was expressed in 95% confidence interval. All statistical analysis was performed using SPSS 22.0 (IBM, New York, United States).

## Results

### Patient and Aneurysm Characteristics

**FIGURE 2:**
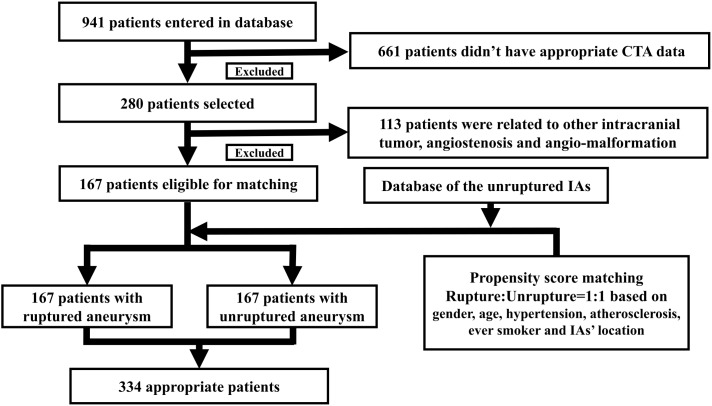
The selecting flow chart was presented here. A total of 941 patients were enrolled into our study. 167 patients with ruptured IA met our standards in our series. Another 167 patients with unruptured IA were then selected using the propensity score matching. The matching process yielded 167 patients in each group.

A total of 941 patients were enrolled into our study. 167 patients with ruptured IA met our standards in our series. Another 167 patients with unruptured IA were then selected using the PSM (selecting flow chart was given as **Figure 2**). Finally, a case-control group, which included 334 patients (167 patients with rupture IA and 167 patients with unruptured IA) was created. The female rate was 55.1% in patients with rupture aneurysms and 55.7% for patients with unruptured aneurysms, respectively. Mean age was 52.3 for ruptured group and 53.9 for unruptured group, respectively. The difference in hypertension history, atherosclerosis history, and smoking history were equal between ruptured and unruptured IAs. No significant difference was found in family history of non-traumatic subarachnoid history (*p* = 0.247). The demographic information was summarized in **Table 1**.

**Table 1:** Demography information.

	Rupture IAs	Unrupture IAs	*p*
	
Characteristics	*n* = 167	*n* = 167	Value
**Gender**			–
Male	75(44.9%)	74(44.3%)	
Female	92(55.1%)	93(55.7%)	
**Mean age (years)**	52.3	53.9	–
**Hypertension history**			–
YES	100(59.9%)	98(58.7%)	
NO	67(40.1%)	69(41.3%)	
**Atherosclerosis history**			–
YES	87(50.9%)	90(53.9%)	
NO	80(49.1%)	77(46.1%)	
**Ever-or-now smoker**			–
YES	94(56.3%)	98(58.7%)	
NO	73(43.7%)	69(41.3%)	
**Family history of non-traumaticsubarachnoid hemorrhage**			0.247
YES	3(1.8%)	1(0.6%)	
NO	164(98.2%)	166(99.4%)	

### Radiological and Morphological Differences Between Ruptured and Unruptured Aneurysms

**FIGURE 3:**
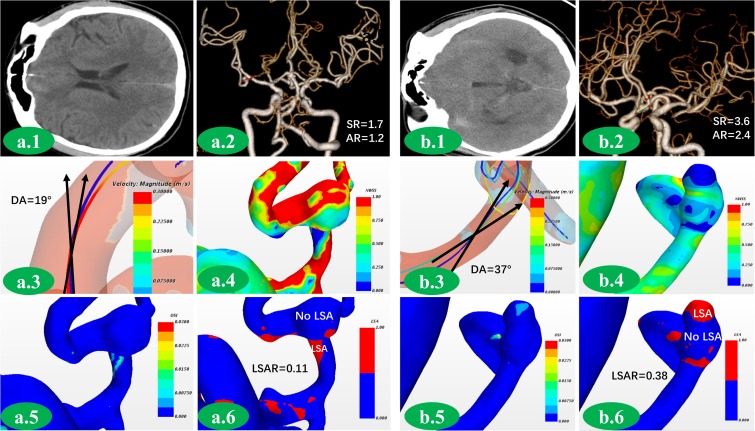
A group of aneurysms with similar match score were presented here. This was an unruptured middle cerebral artery aneurysm **(a.1,2)**. SR was 1.7. AR was 1.2 **(a.2)**. DA was 19°**(a.3)**. NWSS of aneurysm was close to that of parent artery **(a.4)**. There was no area with increasing OSI **(a.5)**. There was no obvious low WSS area in this aneurysm **(a.6)**. This was a ruptured anterior communication artery aneurysm **(b.1,2)**. DA, SR and AR were 37°, 3.6, and 2.4, respectively, which were much higher than unrupture aneurysm **(b.2,3)**. NWSS was lower comparing to parent artery **(b.4)**. Area with obviously increasing OSI could be found **(b.5)**. Large area with low WSS could be found in the dome of aneurysm **(b.6)**.

The ruptured and unruptured group IAs greatly different in the morphological parameters including L(*p* = 0.021), H (*p* < 0001), DA (*p* < 0.001), AR (*p* < 0.001), SR (*p* < 0.001), UI (*p* < 0.001), EI (*p* = 0.002), and NSI (*p* < 0.001) (Radiological and Morphological characteristics were given in **Table 2**). Yet no significant difference was found in D (*p* = 0.082), d (*p* = 0.434), P (*p* = 0.602), volume (*p* = 0.513), and daughter sac (*p* = 0.466). The morphological-hemodynamic analysis for a couple of aneurysms (including a ruptured aneurysm and an unruptured aneurysm) were shown in **Figures 3a,b**.

**Table 2:** Radiological and morphological differences, and hemodynamic differences.

	Rupture IAs	Unrupture IAs	*p*
	
Characteristics	*n* = 167	*n* = 167	Value
**Radiological and morphological characteristics**			

**L (mm)**	**13.82 ± 1.60**	**9.97 ± 1.50**	**0.021**
D (mm)	12.33 ± 3.36	10.61 ± 4.28	0.082
d (mm)	8.55 ± 2.99	7.95 ± 3.04	0.433
**H (mm)**	**11.89 ± 2.26**	**7.81 ± 1.89**	**<0.001**
P (mm)	4.71 ± 1.20	4.61 ± 1.14	0.602
Volume (mm^3^)	4247.08 ± 422.21	4371.82 ± 407.82	0.513
**DA (°)**	**31.07 ± 1.62**	**58.96 ± 1.63**	**<0.001**
**AR**	**1.75 ± 0.07**	**1.21 ± 0.09**	**<0.001**
**SR**	**2.71 ± 0.11**	**1.80 ± 0.15**	**<0.001**
**UI**	**0.08 ± 0.07**	**0.10 ± 0.09**	**<0.001**
**EI**	**0.16 ± 0.13**	**0.10 ± 0.07**	**0.002**
**NSI**	**0.23 ± 0.09**	**0.16 ± 0.08**	**<0.001**
Daughter sac			0.466
YES	46(27.5%)	37(22.2%)	
NO	121(72.5%)	130(77.8%)	
Location			–
Anterior cerebral artery	5(3.0%)	5(3.0%)	
Posterior circulation	9(5.4%)	9(5.4%)	
Middle cerebral artery	41(24.6%)	43(25.7%)	
Anterior communicating artery	48(28.7%)	48(28.7%)	
Internal carotid artery	64(38.3%)	62(37.2%)	
Ophthalmic artery	12	12	
Posterior communicating artery	24	22	
Other	28	28	

**Hemodynamic characteristics**			

Pm(Pa)	2821.89 ± 196.73	2848.94 ± 523.63	0.790
Pa(Pa)	1286.62 ± 130.13	1667.86 ± 188.52	0.626
NPa	0.44 ± 0.18	0.42 ± 0.24	0.569
NPm	0.93 ± 0.45	0.91 ± 0.53	0.490
WSSm(Pa)	5.23 ± 1.31	4.82 ± 2.11	0.511
WSSa(Pa)	1.79 ± 0.21	3.32 ± 0.25	0.350
**NWSSa**	**0.21 ± 0.14**	**0.52 ± 0.22**	**<0.001**
**NWSSm**	**0.59 ± 0.39**	**0.64 ± 0.41**	**<0.001**
**WSSG (Pa/m)**	**14.13 ± 1.014**	**9.15 ± 0.63**	**<0.001**
**LSAR**	**0.39 ± 0.29**	**0.22 ± 0.31**	**0.031**
**OSI**	**0.014 ± 0.012**	**0.0062 ± 0.0022**	**<0.001**
**RRT**	**7.12 ± 4.61**	**4.59 ± 2.74**	**<0.001**

*Bold values indicate statistically significant parameters.*

### Hemodynamic Differences Between Ruptured and Unruptured Aneurysms

Statistical differences were found in hemodynamic parameters including NWSSa (*p* < 0.001), NWSSm (*p* < 0.001), WSSG (*p* < 0.001), LSAR (*p* = 0.031), OSI (*p* < 0.001), and RRT (*p* < 0.001). Yet no significant difference was found for Pm (*p* = 0.790), Pa(*p* = 0.626), NPa (*p* = 0.490), NPm (*p* = 0.490), WSSm (*p* = 0.511), and WSSa (*p* = 0.350). Hemodynamics information was given in **Table 2**.

### Univariate Analysis and Multiple Logistic Regression Analysis

**FIGURE 4:**
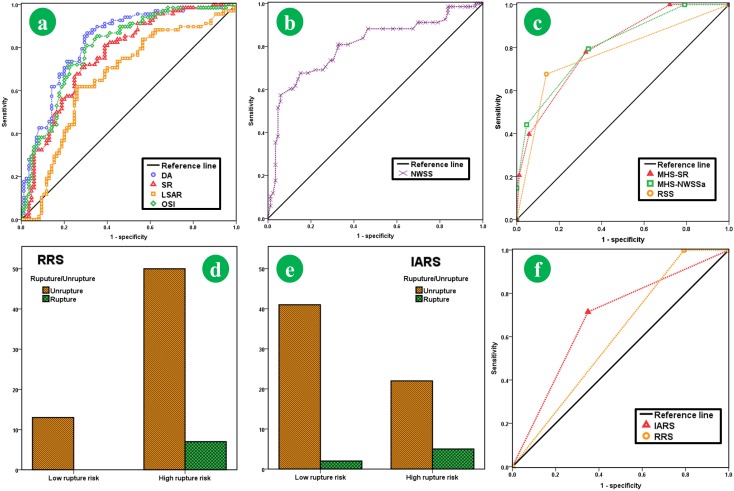
The ROC curves of key parameters (DA, SR, LSAR, OSI, and NWSS) were presented here **(a,b)**. The cut-off values for SR, DA, NWSSa, LSAR, OSI, and RRT were 2.3, 35, 0.24, 0.30, 0.008, and 5.3, respectively. The ROC curves of the IARS-SR, IARS-NWSS, and RRS suggested that the AUC of IARS-SR (AUC 0.81) was larger than IARS-NWSS (AUC 0.79) and RRS (AUC 0.77), which suggested that the IARS-SR had higher accuracy to discriminate the ruputre statue **(c)**. For the follow-up group, the distributions of the ruptured aneurysm and unruptured aneurysm in each stratification system were presented **(d,e)**. The IARS has higher AUC (AUC for the IARS and the RRS was 0.723 and 0.673, respectively.

As suggested by ROC curve analysis, the cut-off values for L, H, AR, SR, EI, UI, NSI, DA, NWSSa, NWSSm, WSSG, LSAR, OSI, and RRT were 13.5, 12.4, 1.6, 2.3, 0.11, 0.13, 0.17, 35, 0.24, 0.78, 15.0, 0.30, 0.008, and 5.3, respectively (AUC, confidence interval and cut-off value were given in **Table 3**, the curves of ROC of several key parameters were presented as **Figures 4a,b**). Univariate regression analysis suggested that AR(*p* = 0.011), SR(*p* < 0.001), DA (*p* < 0.001), EI (*p* = 0.028), NWSSa (*p* < 0.001), NWSSm (*p* = 0.027), LSAR (*p* = 0.003), and OSI (*p* = 0.007) were the risk factors for aneurysm rupture (**Table 3**). Yet no significant difference of L (*p* = 0.344), H (*p* = 0.172), UI (*p* = 0.056), WSSG (*p* = 0.426), and RRT (*p* = 0.059) was shown between the ruptured and the unruptured group. Multivariable regression analysis identified SR (*p* = 0.001), DA (*p* < 0.001), NWSS (*p* = 0.032), LSAR (*p* = 0.007), and OSI (*p* = 0.032) as independent risk factors related to rupture condition (**Table 3**).

**Table 3:** The result of ROC analysis and Logistic regression analysis.

	AUC	Confidence interval	*p*	Cut-off value
**L**	**0.673**	**0.582–0.763**	**0.001**	**13.5**
**H**	**0.750**	**0.673–0.828**	**<0.001**	**12.4**
**AR**	**0.847**	**0.760–0.897**	**<0.001**	**1.6**
**SR**	**0.824**	**0.754–0.893**	**<0.001**	**2.3**
**EI**	**0.768**	**0.692–0.843**	**<0.001**	**0.11**
**UI**	**0.691**	**0.608–0.775**	**<0.001**	**0.13**
**NSI**	**0.877**	**0.820–0.935**	**<0.001**	**0.17**
**DA**	**0.862**	**0.807–0.941**	**<0.001**	**35°**
**NWSSa**	**0.859**	**0.779–0.910**	**<0.001**	**0.24**
**NWSSm**	**0.784**	**0.720–0.869**	**<0.001**	**0.38**
**WSSG**	**0.702**	**0.618–0.796**	**0.004**	**15.0**
**LSAR**	**0.749**	**0.584–0.773**	**<0.001**	**0.30**
**OSI**	**0.843**	**0.660–0.789**	**<0.001**	**0.008**
**RRT**	**0.654**	**0.499–0.761**	**<0.001**	**5.3**

**Variable**	**OR**	**Univariate logistic regression *p* value**	**OR**	**Multivariate logistic regression *p* value**

L	1.40	0.344	–	–
**AR**	**1.63**	**0.011**	11.72	0.053
**SR**	**12.59**	**<0.001**	**3.46**	**0.001**
H	1.70	0.172	–	–
**DA**	**5.86**	**<0.001**	**4.42**	**<0.001**
**EI**	**3.92**	**0.028**	12.76	0.071
UI	19.84	0.056	–	–
**NSI**	**7.90**	**<0.001**	3.17	0.072
WSSG	2.04	0.426	–	–
**NWSSa**	**27.76**	**<0.001**	**0.58**	**0.032**
**NWSSm**	**0.087**	**0.027**	0.03	0.487
**LSAR**	**2.87**	**0.003**	**3.65**	**0.007**
**OSI**	**1.85**	**0.007**	**0.895**	**0.032**
RRT	4.32	0.059	–	–

*Bold values indicate statistically significant parameters.*

### Discrimination Effectiveness and Evaluation of Different Models

Several parameters (SR, DA, NWSS, LSAR, and OSI) were selected in accordance with the regression model analysis (the result of multivariate Logistic regression analysis for the IARS and the RRS was presented in **Supplementary Table 2**). NWSS was found to be strong collinearity with SR (Pearson correlation coefficient = 0.942, *p* < 0.001) in line with result of Pearson correlation. Thus, two models called IARS-SR and IARS-NWSS were separately built (**Table 4**). Then, the discrimination effectiveness of different models was compared by using the ROC analysis. The IARS-SR system had highest discriminating accuracy (AUC 0.81, 95% CI 0.74–0.88, *p* < 0.001), in comparison with IARS-NWSS (AUC 0.79 95% CI 0.69–0.84) and RRS (AUC 0.77 95% CI 0.69–0.85) (AUC and *p*-value were given in **Table 4**; result of ROC was presented in **Figure 4c**). Then, the IARS-SR was defined as IARS system. Herein, **IARS ≥ 2** was defined as the high risk group.

**Table 4:** Intracranial Aneurysms Rupture Score (IARS) and result of ROC of three models.

Score	0	1
**IARS-SR**		
SR	<2.3	>2.3
DA(°)	<35°	>35°
LSAR	<0.3	>0.3
OSI	<0.008	>0.008
**IARS-NWSSa**		
NWSSa	>0.24	<0.24
DA(°)	<35°	>35°
LSAR	<0.3	>0.3
OSI	<0.008	>0.008

**ROC analysis**	**AUC**	***p*-value**

**IARS-SR**	**0.809**	**<0.001**
**IARS-NWSSa**	**0.778**	**<0.001**
**RRS**	**0.767**	**<0.001**

*Bold values indicate statistically significant parameters.*

### Further Validation

**FIGURE 5:**
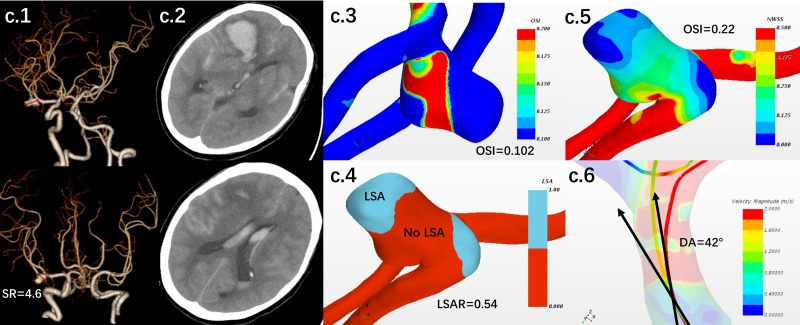
A female patient with middle cerebral artery aneurysm was followed up by clinical visiting. The patient had hypertension history. Based on the first CTA, SR, DA, LSAR, and OSI were 4.6, 42°, 0.74 and 0.22, respectively **(c.1,3,4,5,6)**. The aneurysm was scored by the IARS as 4, which was stratified as high-risk group. 3.5 months later, the aneurysm was rupture. SAH and hemotoma were confirmed by CT **(c.2)**.

Two hundred and twenty one patients visited in the outpatient for IA in total. At the end of following up, 7 patients (the rupture rate was 1.6% per year in our series) were subject to IAs rupture. The matching process yielded 70 appropriate patients (included 7 ruptured IAs and 63 unruptured IAs, the hemodynamic-morphological characteristics of the ruptured IAs were given in **Table 5**). Demography, morphological, and hemodynamic information for each patient were given in **Supplementary Table 1**. No statistically significant difference was found in gender, age, hypertension, atherosclerosis, now or ever smoker, and aneurysm location. Stratified by IARS, 5/26 patients in high risk group (19.2%) undergoing SAH or intracranial hemorrhage, caused by IAs, and only 2/44 patients in low risk group (4.55%) were subject to that during the follow-up period. Yet stratified by RRS, 7/57 patients in high risk group (12.3%) were subject to SAH or intracranial hemorrhage, and 0/13 patients in low risk group (0.0%) were subject to that (a hemodynamic-morphological analysis for a ruptured IA was shown in **Figure 5**). The discriminating accuracy is significantly different between two score system (19.2% vs. 12.3%). As revealed by ROC analysis, the IARS has higher AUC (AUC for the IARS and the RRS was 0.723 and 0.673, respectively, **Figures 4d–f**). The result of ROC and rupture rate was summarized in **Table 5**.

**Table 5:** Hemodynamic and morphological characteristics of rupture IAs.

Patients	Time from finding to rupture (month)	SR	DA (°)	LSAR	b	NWSSa	Stratification by RRS	Stratification by IARS
1	3.5	4.6	42	0.54	0.010	0.22	High risk	High risk (4)
2	4.9	3.2	36	0.51	0.007	0.21	High risk	High risk (3)
3	7.2	2.9	22	0.43	0.007	0.16	High risk	High risk (2)
4	6.3	2.6	39	0.39	0.022	0.15	High risk	High risk (4)
5	10.4	2.1	18	0.49	0.005	0.48	High risk	Low risk (1)
6	5.9	3.3	38	0.32	0.014	0.14	High risk	High risk (4)
7	6.2	3.2	14	0.28	0.007	0.38	High risk	Low risk (1)

		**Ruptured IAs at high-risk group**					**Unruptured IAs at low risk group**	
		
IARS		5/26(19.2%)					42/44(95.5%)	
RRS		7/57(12.3%)					13/13(100.0%)	

**Result of ROC analysis**

		**AUC**					**Confidence interval**	
IARS		0.723					0.576–0.889	
RRS		0.673					0.413–0.793	

## Discussion

Some clinical trials have tried to establish a method to assess rupture risk of IAs, such as the International Study of Unruptured Intracranial Aneurysms study and the PHASES study (Greving et al., 2014). Yet models established in those studies included only clinical factors, which were difficult to be explained by the internal mechanisms of IAs rupture. Recent studies suggested that the hemodynamic and morphological characteristics of aneurysm were also important to assess rupture risk. In this study, by using a PSM matched IA data base of 334 aneurysms from our institution, we attempted to find out hemodynamic and morphological characteristics of ruptured IAs and establish a useful score system to help clinical work.

Following the previous studies, we balanced the baseline and excluded the known risk factors by using the PSM. In the International Study of Unruptured Intracranial Aneurysms study, author reported that different location had different rupture rate (Wiebers, 2006). Previous Varble et al. (2017) found that internal carotid aneurysms are the least rupture-prone. Thus, location is an important risk factor to assess rupture risk. By using pool analysis, the PHASES study demonstrated population, hypertension, age, SAH history, and site of aneurysm as independent risk factors for aneurysm rupture (Greving et al., 2014). Here, we balanced those risk factors between ruptured and unruptured IAs by using PSM. This statistical method could exclude the confounding factors.

SR is relative to the parent vessel, has been independently associated with rupture risk in many studies. High SR, combined with abnormal hemodynamic conditions, may result in an aneurysm rupture. Previous CFD studies have found that with increasing SR, aneurysm rupture risk increased. However, previous Meng et al. reported that a SR > 1.75 could increase the rupture risk (Xiang et al., 2011). In another study, Dhar et al. (2008) reported that a SR > 2.05 was a risk factor for IA rupture. Based on our data, we found that cut-off value between ruptured and unruptured IAs was 2.3, which was larger than previous study. The finding is not surprising because previous many studies calculated this parameter based on the image data after rupture. However, a study analyzed the morphological change before and after aneurysm rupture and gave the conclusion that postrupture morphology should not be considered to assess the rupture risk (Skodvin et al., 2017b). It is essential to take this factor into consideration.

Low WSS has been previously relative to IA rupture. Chien et al. (2009) found that low WSS was associated IA rupture in poster circulation aneurysms and AcomA aneurysms. Miura et al. (2013) demonstrated low WSS as an independent risk factor for IA rupture. By a PHASES-based study, Varble et al. (2017) found the IAs other than the ICA are subjected to a low WSS hemodynamic condition that may lead to IA rupture. Thus, WSS, the frictional force between blood and aneurysm wall, could use as a parameter to assess the rupture risk. The other important parameter is LSAR. The friction between blood and endothelial cells is essential for normal arterial wall proliferation. Low or stagnant flow can result in an inflammatory response in the vascular wall (Meng et al., 2014). This phenomenon was found in the ruptured aneurysm wall as well as apoptosis and degeneration of the matrix (Cebral et al., 2017). Thus, larger low WSS area suggested severe injury for aneurysm wall. In 36 aneurysms, previous study found LSAR was associated with aneurysm rupture by using case-control study (Varble et al., 2017). Our data reflect a similar result, that aneurysm with low WSS and high LSAR have a high risk of incurring an aneurysm rupture. However, there is a difference comparing to result from previous study. Previous Meng et al. has reported that NWSSa in rupture aneurysm was 0.33 ± 0.28 and suggested that a cut-off value of NWSSa was 0.39, which were quite higher than our data (Xiang et al., 2011). In another study, NWSSa was 0.327 ± 0.181 (Qiu et al., 2017). Our data suggested a similar result, that NWSSa in ruptured aneurysm is 0.21 ± 0.14 and the cut-off value of NWSSa is 0.24. Meanwhile Meng et al. also reported LSAR in ruptured aneurysm was 0.38 ± 0.31 and cut-off value was 0.21 (Xiang et al., 2011). However, in a case-control study, the author found a LSAR in ruptured aneurysm was 0.15 ± 0.16 (Skodvin et al., 2017a). In our series, LSAR in ruptured aneurysm was 0.39 ± 0.29 which was similar to Meng et al., but we suggested a cut-off value of LSAR was 0.30 which was much larger than before. For this, we thought a similar explanation of SR may apply to NWSSa and LSAR. However, the real threshold may need a further exploration. OSI was also considered in many previous studies. High OSI is known to upregulate endothelial surface adhesion molecules, which can cause dysfunction of nitrous oxide and inflammatory cell infiltration (Malek et al., 1999). Many studies demonstrated it as an independent risk factor for aneurysm rupture (Xiang et al., 2011; Qiu et al., 2017). Previous study found that OSI in ruptured aneurysms was 0.016 ± 0.031 (Xiang et al., 2011). Our data have similar result, and it is useful to assess the rupture risk.

A novel parameter called DA was further identified, which was defined as the angle between the normal vector and co-velocity. The deviation of blood stream from the vascular center and the possibility of impact effect of vascular wall could be reflected by DA. There have been some studies that have confirmed the role of the impact effect in occurrence and rupture of aneurysms (Omodaka et al., 2012; Zhang et al., 2014; Cebral et al., 2015a). Cebral et al. (2017) reported flow impaction could cause increased stress in the aneurysmal wall where the flow is typically lower, which may lead to the endothelia injury. Those areas are usually presented as low WSS. Malek et al. (1999) found biological responses of the endothelium to low WSS. Also, several studies have confirmed that endothelial cells are damaged by blood flow with low WSS and high OSI, which activates the inflammation or results in wall remolding and atherosclerosis (Malek et al., 1999; Hashimoto et al., 2006). Thus, the deviated blood stream, which could reflect by DA, could damage the vascular endothelia and lead to catastrophic outcome. By considering this, the interaction between blood stream and vascular wall can be understood more thoroughly.

Yet results from many recent studies found that there were many other risk factors contribute significantly to IAs rupture, which were not involved in RRS system. The main reason why RRS did not include these risk factors was that the aneurysm studied was the ruptured aneurysm. After the rupture, the morphology and hemodynamic characteristics of the aneurysm will change dramatically (Skodvin et al., 2017b). Thus, although RRS can already effectively discriminate ruptured and unruptured aneurysms, the accuracy of it could be further increased. As described above, those significant parameters were not totally included in our model because NWSSa suggested a strong collinearity with SR, which should be considered. A study found that NWSSa and OSI are easy to resolve and have good convergence (Evju et al., 2017). Thus, they are suitable to use as discriminating parameters. Accordingly, we established two models in accordance with SR and NWSS, respectively, and confirmed that the IARS-SR has the highest accuracy. Till now, many studies supposed the idea that combining hemodynamic and morphological characteristics is closely related to IAs rupture (Omodaka et al., 2012; Zhang et al., 2014; Cebral et al., 2015a,b). Thus, considering both hemodynamic and morphological factors can thoroughly understand the mechanism of IAs rupture. That is the reason why RRS can effectively discriminate IAs rupture. Yet recent many studies propose other parameters including LSAR, which RSS was not involved, could also suggest the abnormal characteristics (Zhang et al., 2016a,b). In line with recent studies, SR, NWSSa, and OSI, in which RSS is considered, cannot just reflect the possible area of vascular injury and impact effect to vascular wall (Castro, 2013; Chen et al., 2016; Paliwal et al., 2017). In this study, by following up a group of unruptured patients, the unruptured ratio in low risk group was 100% stratified by the RRS but 95.5% stratified by the IARS. However, the ruptured rate in high risk group was 19.2% stratified by the IARS but 12.3% stratified by the RRS. Further ROC analysis was also suggested that the IARS had higher accuracy in discriminating rupture IAs here (AUC for the IARS and the RRS was 0.723 and 0.673, respectively). Meanwhile, if a tool can effectively help clinical work, it should be easy to understand and simple operation. Our study developed a score system to simplify and visualize the morphological-hemodynamic analysis of aneurysm. The proposed model is meant to help physicians who are contemplating timely intervention in face of IA patients.

## Limitations

There are some limitations in our study. First and foremost, as this study is a retrospective study, small sample and short-time follow-up, the conclusion is limited and needs further study to provide more evidences to confirm value of the IARS. Second, although we used the image before rupture to avoid the effect from the event of the rupture, which might not completely reflect the rupture condition. However, the considered statue was nearly close to rupture condition. It may need further prospective study to explore that. Third, we just balanced the risk factors based on previous studies here. However, there may be potential risk factors which may affect the hemodynamic condition in aneurysm. Thus, it was difficult to avoid selection bias because of high selection (we selected 167 from 941 patients with ruptured aneurysm). Fourth, all morphological parameters were calculated from 3D CTA which is suitable for clinical visiting and can exclude the effect from thrombus, but do not give essentially image like 3D angiography. Meanwhile, all hemodynamic models were established from 3D CTA that may affect the accuracy of our simulation. Correlation with angiogram can improve the result, but we did not routinely perform angiogram in clinical visiting. Fifth, our threshold of some parameters was different with previous study. However, all aneurysms in our series came from a single center. The real threshold may require pooling of data from multiple centers. Sixth, some clinical characteristics were not considered, such as multi-IAs and IAs with vascular stenosis.

There are also several limitations related to our CFD analysis. We used the similar modeling and simulating method from Meng et al. The limitations that Meng have given will also exist in our study, such as the traction-free boundary condition at the outlet and definition of low WSS. In our study, differently from Meng, we used the velocity inlet boundary from a representative patient. However, the velocity boundary does not match with the CTA which was scanned before aneurysm rupture. Thus, the pulsatile wave may not be a good representation of the ruptured condition. Considering the CFD is sensitive to the velocity condition, it may affect our result. In the other hand, the effects from magnitude of wave form and so on will also limit our result. In the future study, we may invite reliable ultrasonic expert to join our study. The parameters in the IARS (SR, NWSSa, and OSI) are relatively easy to resolve. However, strict convergence in traditional sense is difficult to reach. Our effort was done to reach the better result. All those questions will be solved in the further study. However, multiple research groups are trying to use CFD analysis to assess aneurysm rupture risk. We believe that CFD may help physicians to make decision-making in the future.

## Conclusion

SR, DA, NWSS, LSAR, and OSI were independent risk factors related to IAs rupture. In comparison with RRS system (including SR, NWSS, and OSI), our present IARS system (including SR, DA, LSAR, and OSI) had higher accuracy in discriminating the rupture status of IAs.

## Ethics Statement

The study was approved by the institutional review board. Written informed consents were obtained from all participants or their legally authorized representatives and privacy of patients was effectively protected.

## Author Contributions

SW and YC were in charge of supervising the whole study. PJ and QL were responsible for the design of the article and drafting. QL was responsible for revising, responding to reviewer and statistical analysis. PJ and JW were also responsible for statistical analysis and confirmed the final article. ML, ZL, SY, and RG were responsible for collecting data. BG and YC provided a reliable technical support.

## Conflict of Interest Statement

The authors declare that the research was conducted in the absence of any commercial or financial relationships that could be construed as a potential conflict of interest.

## Abbreviations

AR: aspect ratio
AUC: area under the curve
CFDs: computational fluid dynamics
CTA: compute tomography angiography
DA: deviated angle
IAs: intracranial aneurysms
LSAR: low shear area ratio
NPa: normalized average pressure
NPm: normalized maximal pressure
NWSSa: normalized average wall shear stress
NWSSm: normalized maximal wall shear stress
OSI: oscillatory shear index
ROC: receiver operating characteristic
RRT: relative resident time
SAH: subarachnoid hemorrhage
SR: size ratio
WSS: wall shear stress
WSSG: wall shear stress gradient.
