# Supplementary material for: A Novel Scoring System for Rupture Risk Stratification of Intracranial Aneurysms: A Hemodynamic and Morphological Study
Source: Front Neurosci. 2018 Sep 5;12:596. doi: 10.3389/fnins.2018.00596 (PMC6133991; doi:10.3389/fnins.2018.00596)
Supplement: Supplementary file 1 [file Data_Sheet_1.docx]

| **Supplementary Table 1. Demography information of follow-up patients** | | |  |
| --- | --- | --- | --- |
|  | Rupture IAs | Unrupture IAs | p |
| Characteristics | n=7 | n=63 | Value |
| Gender |  |  | - |
| Male | 3(42.9%) | 24(38.1%) |  |
| Female | 4(57.1%) | 39(61.9%) |  |
| Mean age (years) | 56.4 | 49.7 | - |
| Hypertension history |  |  | - |
| YES | 3(42.9%) | 39(61.9%) |  |
| NO | 4(57.1%) | 24(38.1%) |  |
| Atherosclerosis history |  |  | - |
| YES | 3(42.9%) | 26(41.3%) |  |
| NO | 4(57.1%) | 37(58.7%) |  |
| Ever-or-now smoker |  |  | - |
| YES | 4(57.1%) | 42(66.7%) |  |
| NO | 3(42.9%) | 21(33.3%) |  |
| Family history of nontraumatic subarachnoid hemorrhage |  |  |  |
| YES | 0(0.0%) | 0(0.0%) | 1.000 |
| NO | 7(100.0%) | 63(100.0%) |  |

| **Supplementary Table 2. The result of multivariate Logistic regression for the IARS and the RRS** | | | | | |
| --- | --- | --- | --- | --- | --- |
| Variables | IARS | |  | RSS | |
|  | OR | p |  | OR | p |
| **SR** | **3.46** | **0.001** |  | **2.28** | **0.020** |
| **DA** | **4.42** | **<0.001** |  | **-** | **-** |
| **NWSSa** | **0.58** | **0.032** |  | **1.87** | **0.044** |
| **LSAR** | **3.65** | **0.007** |  | **-** | **-** |
| **OSI** | **0.895** | **0.032** |  | **2.29** | **0.021** |
